# Wiskott-Aldrich syndrome protein regulates autophagy and inflammasome activity in innate immune cells

**DOI:** 10.1038/s41467-017-01676-0

**Published:** 2017-11-17

**Authors:** Pamela P. Lee, Damián Lobato-Márquez, Nayani Pramanik, Andrea Sirianni, Vanessa Daza-Cajigal, Elizabeth Rivers, Alessia Cavazza, Gerben Bouma, Dale Moulding, Kjell Hultenby, Lisa S. Westerberg, Michael Hollinshead, Yu-Lung Lau, Siobhan O. Burns, Serge Mostowy, Mona Bajaj-Elliott, Adrian J. Thrasher

**Affiliations:** 1 Infection, Immunity and Inflammation Program, Great Ormond Street Institute of Child Health, University College London, 30 Guilford Street, London, WC1N 1EH UK; 2 Department of Paediatrics and Adolescent Medicine, LKS Faculty of Medicine, The University of Hong Kong, Hong Kong, SAR China; 3 Section of Microbiology, MRC Centre of Molecular Bacteriology and Infection, Imperial College London, Armstrong Road, London, SW7 2AZ UK; 4 University College London Institute of Immunity and Transplantation, London, NW3 2PF UK; 5 Karolinska Institutet, Department of Laboratory Medicine, 14186 Stockholm, Sweden; 6 Karolinska Institutet, Department of Microbiology, Tumor and Cell Biology, 171 77 Stockholm, Sweden; 7 Department of Pathology, University of Cambridge, Tennis Court Road, Cambridge, CB2 1AP UK; 8 Shenzhen Primary Immunodeficiency Diagnostic and Therapeutic Laboratory, The University of Hong Kong-Shenzhen Hospital, Shenzhen, China; 9 Great Ormond Street Hospital NHS Foundation Trust, Great Ormond Street, London, WC1N 3JH UK

## Abstract

Dysregulation of autophagy and inflammasome activity contributes to the development of auto-inflammatory diseases. Emerging evidence highlights the importance of the actin cytoskeleton in modulating inflammatory responses. Here we show that deficiency of Wiskott–Aldrich syndrome protein (WASp), which signals to the actin cytoskeleton, modulates autophagy and inflammasome function. In a model of sterile inflammation utilizing TLR4 ligation followed by ATP or nigericin treatment, inflammasome activation is enhanced in monocytes from WAS patients and in WAS-knockout mouse dendritic cells. In ex vivo models of enteropathogenic *Escherichia coli* and *Shigella flexneri* infection, WASp deficiency causes defective bacterial clearance, excessive inflammasome activation and host cell death that are associated with dysregulated septin cage-like formation, impaired autophagic p62/LC3 recruitment and defective formation of canonical autophagosomes. Taken together, we propose that dysregulation of autophagy and inflammasome activities contribute to the autoinflammatory manifestations of WAS, thereby identifying potential targets for therapeutic intervention.

## Abstract (web summary)

Wiskott-Aldrich syndrome protein (WASp) is essential for controlling the cytoskeleton, but its function in innate immunity is unclear. Here the authors show that WASp deficiency is associated with dysregulated septin cage formation, excessive inflammasome activation, elevated immune cell death and reduced bacterial clearance.

## Introduction

Wiskott–Aldrich syndrome (WAS) is an X-linked recessive primary immunodeficiency disorder characterized by microthrombocytopenia, defective immunity and eczema. Autoimmune disorders occur in 20–70% of patients with WAS; common manifestations include autoimmune haemolytic anaemia, neutropenia, vasculitis, arthritis and inflammatory bowel disease^1, 2^. Some features of WAS resemble paradigmatic auto-inflammatory syndromes, but underlying mechanisms have not been explored. Monogenic autoinflammatory disorders are characterized by mutations that result in overt caspase-1 activation, which consequently promotes exaggerated bioactive cytokine (interleukin-1β (IL-1β) and IL-18) secretion and pyroptosis, a form of inflammatory cell death^3^. Extensive research has identified a family of inflammasome complexes as important regulators of these cellular events^4–7^. Danger/stress signals generated in response to infection and/or inflammation are ‘sensed’ by innate sensors. Among the nucleotide-oligomerization domain and leucine rich-repeat containing (NLR) family members, NLRP3 is considered a promiscuous sensor as it can activate the inflammasome in response to a diverse range of soluble and particulate stress signals, including ATP and silica^8^. NLRP3 activation results in the recruitment of an adapter protein, apoptosis-associated speck-like protein containing a CARD (ASC), and downstream docking of pro-caspase-1. NLRP3/ASC/pro-caspase-1 complex formation promotes autocatalytic activation of pro-caspase-1 to caspase-1, which in turn processes pro-IL-1β/pro-IL-18 to their secretory, bioactive forms^9^.

Toll-like receptor (TLR)-mediated, nuclear factor-κB (NF-κB)-driven transcriptional upregulation of sensor molecules (including NLRP3/NLRC4), as well as pro-IL-1β and pro-IL-18, generally precedes inflammasome activation^10, 11^. TLR-mediated gene expression is a common host response to commensal and pathogenic organisms alike; activation of this pathway is generally called ‘signal 1’ or ‘priming’. Exogenous noxious agents (such as bacterial toxins) or endogenous danger-associated molecular patterns, such as ATP, generate a second stress response, referred to as ‘signal 2’, which initiates recruitment and activation of the inflammasome complex and immunity^3, 4, 12^.

Evidence indicates that the inflammasome machinery is intimately linked with another intracellular innate defence pathway, namely autophagy^13–16^. Autophagy is an ancient conserved mechanism involved in maintaining nutritional homeostasis that provides immune protection by targeting infectious agents into autophagosomes, which direct loaded cargo to the lysosomal compartment for processing and destruction^16–18^. Bacterial autophagy, also known as xenophagy, is central to directing phagocytosed microbes to lysosomal degradation^16, 18^. Although cytoskeletal rearrangements have a major function in these processes, molecular details are unclear. Studies have demonstrated an integral function for septins, a class of GTP-binding proteins closely associated with the actin cytoskeleton. Septins can form cage-like structures that entrap bacteria and target them to autophagy, thus restricting cytoplasmic replication^19–21^. In addition to being a requirement for septin cage formation, the actin cytoskeleton is an important regulator of inflammasome activation and in shaping the autophagosomal membrane^22–27^.

WAS protein (WASp) is an important regulator of the actin cytoskeleton by modulating Arp2/3-mediated actin polymerization in haematopoietic cells, and thus is important to multiple aspects of immune cell function^2, 28^. In the present study, we show that WASp-mediated actin cytoskeletal rearrangements in innate immune cells are central in regulating autophagy and inflammasome activities in response to both chemical and bacterial stimuli. We found that WASp participates in bacterial septin cage formation, a cellular assembly that affects the inflammasome axis during autophagic destruction of intracellular bacteria. In addition, we showed that WASp has an important function in autophagosome formation for bacterial delivery to the lysosomal compartment.

## Results

### Increased NLRP3 activation in WASp-deficient myeloid cells

**Fig. 1:**
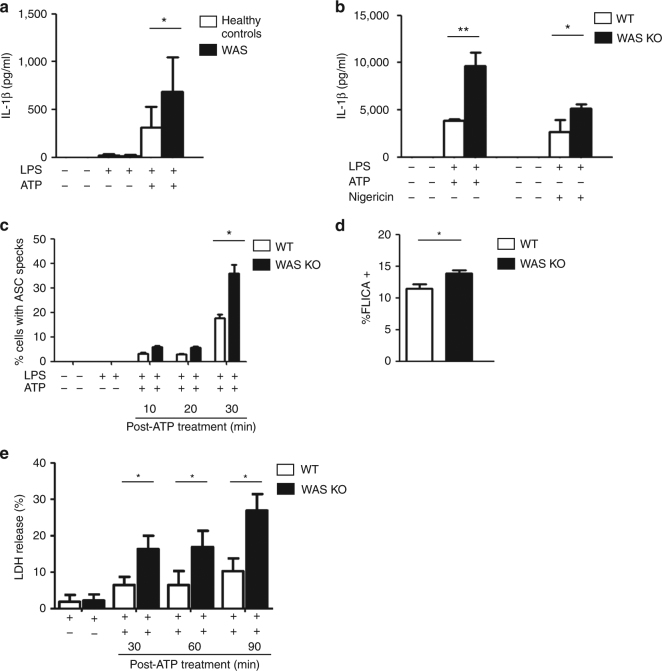
Increased NLRP3 activation in WASp-deficient myeloid cells. **a** CD14^+^ monocytes from healthy controls (*n* = 6) and patients with classical WAS (*n* = 3; Ochs’ score ≥4, and no WASp expression by flow cytometry) were primed with LPS (50 ng/ml) for 3 h, followed by ATP stimulation (3 mM) for 30 min. After stimulation, IL-1β was evaluated in culture supernatants by ELISA. Data presented are mean protein concentration ± SEM performed in duplicates. Two-sided Student’s *t*-test. **b** WT or WAS KO BMDCs were primed with LPS (100 ng/ml) for 3 h, followed by stimulation with ATP (5 mM) or nigericin (10 μM) for 30 min. After stimulation, IL-1β in culture supernatants was quantified by ELISA. Data presented are mean protein concentration ± SEM performed in duplicates from three independent experiments. Two-sided Student’s *t*-test. **c** WT or WAS KO BMDCs were primed with LPS (100 ng/ml) for 3 h and stimulated with ATP (5 mM) for 10, 20 or 30 min. Cells were fixed and stained for nuclei (blue), ASC (green) and F-actin (red). Images were taken by confocal microscopy (×63 magnification). Percentage of BMDCs containing ASC specks was quantified. Results are mean ± SEM from three independent experiments, with at least 200 cells evaluated in each experiment per time-point. Two-sided Student’s *t*-test. Representative confocal images are shown in Supplementary Fig. 1G. **d** WT or WAS KO BMDCs were primed with LPS (100 ng/ml) for 3 h, followed by ATP (5 mM) stimulation for 30 min. Intracellular active caspase-1 was detected by binding of FAM-YVAD-FMK (FAM-FLICA). Cells emitting green fluorescence were detected in the FL-1 gate. Results are mean percent of FLICA^+^ BMDCs ± SEM from three independent experiments each performed in duplicate. Two-sided Student’s *t*-test. **e** WT or WAS KO BMDCs were primed with LPS (100 ng/ml) for 3 h and stimulated with ATP (5 mM) for 30, 60 or 90 min. Time-dependent increase in LDH release was indicative of pyroptotic cell death. LDH measurement upon lysis of control, uninfected cells with 0.1% Triton X-100 was considered 100% release. Data presented represent are mean ± SEM of LDH release from three independent experiments each performed in duplicates. Two-sided Student’s *t*-test. *p < 0.05; **p < 0.01

To investigate the effect of WASp deficiency on inflammasome activity, human peripheral blood CD14^+^ monocytes from six healthy controls and three patients with classical WAS (WASp-null) were primed with lipopolysaccharide (LPS) with or without adenosine triphosphate (ATP) stimulation. LPS-mediated Toll-like receptor 4 (TLR4) ligation triggers events (signal 1) that promote the synthesis of several inflammasome components and pro-IL-1β. ATP and nigericin are considered ‘classical’ triggers of NLRP3-mediated inflammasome activation culminating in cytokine secretion and pyroptosis^7^. LPS alone induced minimal amounts of IL-1β secretion in healthy controls and in WAS; however, upon ATP stimulation WAS monocytes exhibited significantly increased IL-1β secretion; Fig. 1a). We also tested the effect of LPS/nigericin on monocytes from two patients with attenuated WAS (hypomorphic missense mutations, L39R and T35M in the *WAS* gene, respectively), and found that IL-1β secretion in this patient group was comparable to healthy controls (Supplementary Fig. 1A), suggesting that the complete absence of WASp promotes enhanced inflammasome activity.

To gain better understanding of the mechanism(s) involved, wild-type (WT) and WAS-knockout (WAS KO) bone marrow-derived dendritic cells (BMDCs) were exposed to LPS followed by ATP or nigericin; both agonists mediated significant increase in IL-1β secretion in WAS KO BMDCs compared to the WT counterpart (*p* < 0.001, *p* < 0.05, respectively, unpaired Student’s *t*-test; Fig. 1b). We found minimal variation in signal 1 between the two cell types as no significant difference was recorded in (a) LPS-mediated NF-κB activation in WT and WAS KO BMDC (measured by a lentiviral-transduced NF-κB reporter assay; Supplementary Fig. 1B, C) and (b) pro-IL-1β protein expression in response to LPS (Supplementary Fig. 1D). In addition, basal IL-1β receptor expression was found to be similar (Supplementary Fig. 1E). Taken together, our observations indicate that dysregulated inflammasome activation is responsible for the observed increase in IL-1β secretion. In order to confirm the contribution of WASp deficiency to enhanced inflammasome activity, WAS KO BMDCs were transduced with a lentiviral vector expressing enhanced green fluorescent protein (eGFP) fused to human WASp. Although the reconstitution resulted in some diminution of IL-1β production, this did not reach significance, as this was most likely due to the consistently low (10–20%) transduction efficiency achieved.

ATP triggers the formation of a large supramolecular assembly of NLRP3, ASC and procaspase-1^29^. The multimeric ASC aggregates, known as pyroptosomes, can be visualized as ASC ‘specks’. To support our hypothesis that WASp deficiency impacted on the inflammasome axis, we investigated LPS/ATP-mediated speck formation in our BMDC model system. Specks were visible as early as 10 min after ATP stimulation and a statistically significant increase in speck formation was observed in WAS KO BMDCs compared with WT cells within 30 min (Fig. 1c and Supplementary Fig. 1F). Although active caspase-1 was readily detectable in ATP-stimulated WT and WAS KO BMDCs by western blotting (Supplementary Fig. 1G), greater sensitivity offered by flow cytometry revealed a significant difference in caspase-1 activity (as measured by FLICA^+^ cells (Fig. 1d) and western blot (Supplementary Fig. 1H)) in ATP-stimulated WAS KO BMDCs compared with WT cells. Significantly increased cell death (LDH release) was also recorded in the absence of WASp (Fig. 1e).

### Inflammasome hyperactivity after bacterial challenge in WAS

**Fig. 2:**
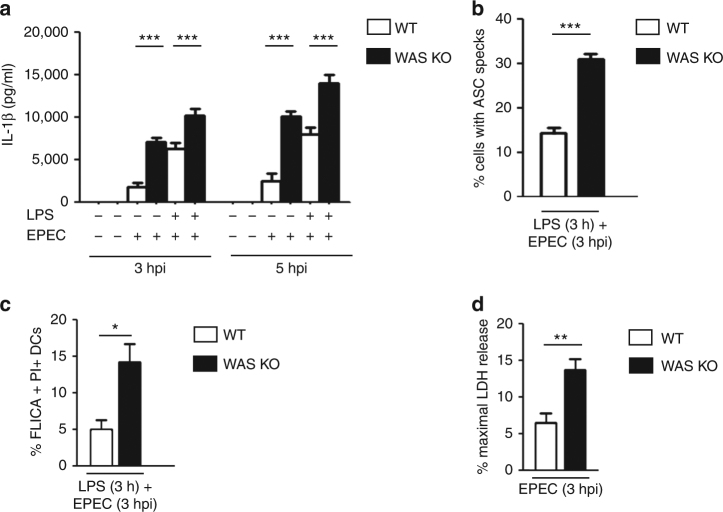
Enteropathogenic *E. coli*-mediated inflammasome activation. **a** WT or WAS KO BMDCs were co-cultured with WT E2348/69 EPEC strain at an MOI of 5 for 3 or 5 h. After co-culture, cells were treated with 100 µg/ml gentamicin for 2 h to kill extracellular bacteria (total 5 or 7 hpi). In parallel experiments, BMDCs were primed with LPS (100 ng/ml) for 3 h prior to infection. After infection, IL-1β protein levels were quantified in culture supernatants by ELISA. Results are mean protein concentration ± SEM of three independent experiments in duplicate. Two-sided Student’s *t*-test. **b** WT or WAS KO BMDCs were primed with LPS (100 ng/ml) for 3 h prior to co-culture with WT EPEC (MOI of 5) for 3 h. After fixation, cells were stained for bacteria (DAPI (4′,6-diamidino-2-phenylindole)), ASC (green) and actin phalloidin (red). Images were taken by confocal microscopy (×40 magnification) and the percentage of BMDCs containing ASC specks were quantified. Results are represented as mean ± SEM from three independent experiments. A minimum of 200 BMDCs were evaluated per quantification. Two-sided Student’s *t*-test. Representative confocal images are shown in Supplementary Fig. 2A. **c** WT or WAS KO BMDCs were primed with LPS (100 ng/ml) for 3 h, followed by co-culture with WT EPEC (MOI of 5) for 3 h. Intracellular active caspase-1 was detected by FAM-YVAD-FMK (FAM-FLICA) binding, dead cells were stained with PI. The percentage of FLICA and PI double-positive BMDCs were enumerated by flow cytometry (FLICA collected in FL-1 gate; PI collected in FL-2 gate). Results are mean ± SEM of three independent experiments. Two-sided Student’s *t*-test. A representative flow plot is shown in Supplementary Fig. 2B. **d** WT or WAS KO BMDCs were co-cultured with WT EPEC (MOI of 5). Three hours after infection, pyroptotic cell death was measured by LDH release, expressed as a percentage of maximum LDH release measured from control, uninfected cells. Results are mean ± SEM of three independent experiments. Two-sided Student’s *t*-test. *p < 0.05; **p < 0.01; ***p < 0.001

Enteropathogenic *E. coli* (EPEC) engages the NLR family to mediate inflammasome activation in murine and human innate immune cells^30, 31^. EPEC also cause major actin cytoskeletal rearrangements within the host cell^32–34^. Herein, we utilized EPEC-BMDC coculture model system of infection to investigate the role of the actin cytoskeleton and WASp in BMDC-mediated inflammasome activation. BMDCs were co-cultured (with or without LPS priming) with the WT E2348/69 EPEC strain for 3 and 5 h. In parallel experiments, cells were also subjected to LPS priming prior to exposure to EPEC (to ensure equal induction of signal 1). A significant difference in cytokine secretion was observed between infected WT and WAS KO BMDCs at 3 and 5 h post-infection (hpi) and this effect was further enhanced in response to LPS priming (Fig. 2a). A statistically significant increase in percentage of ASC specks (exposed to 3 h LPS followed by 3 h infection), and FLICA (caspase-1 active) and propidium iodide (PI) double-positive (pyroptotic) cells in WT vs. WAS KO BMDCs was also recorded (Fig. 2b, c and Supplementary Fig. 2A). As LPS alone showed no differential effect on LDH release between WT and WAS KO BMDCs (Fig. 1e), we focused on the effect of infection alone on cell death. In agreement with the hypothesis that WASp deficiency impacts on the inflammasome axis, infected WAS KO BMDCs exhibited significantly greater pyroptosis than infected WT cells (Fig. 2d and Supplementary Fig. 2B).

### WASp deficiency leads to defective bacterial clearance

Microbial uptake and/or invasion leads to rapid induction of autophagy, a process responsible for capturing and directing phagocytosed and/or cytosolic invaders for lysosomal degradation, processing and antigen presentation^14–16^. Although molecular details are currently lacking, limited evidence indicates that the autophagy and the inflammasome pathways are closely interlinked^14–16, 35^. We hypothesized that the observed increase in IL-1β release in EPEC-infected WAS KO BMDCs compared to infected WT cells is due to impaired intracellular bacterial processing.

**Fig. 3:**
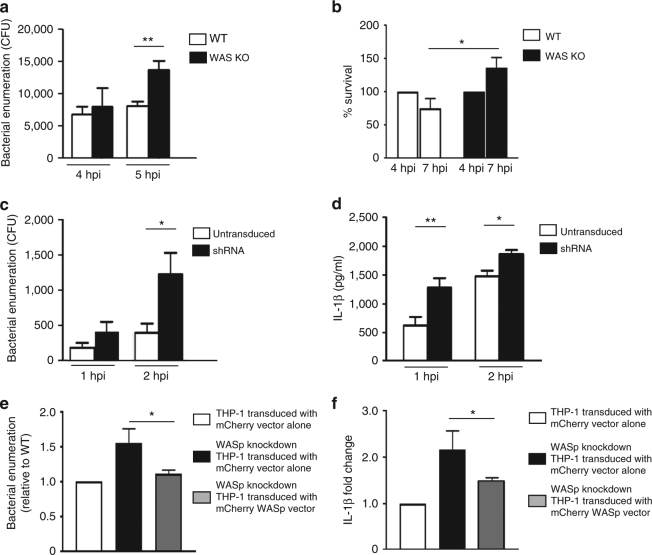
WAS deficiency leads to defective bacterial clearance. **a** BMDCs were co-cultured with WT EPEC (MOI of 5) for 2 or 3 h, followed by a 2 h gentamicin (100 µg/ml) treatment. At 4 or 5 hpi, intracellular live bacteria were enumerated. Data presented are mean CFU ± SEM of three independent experiments in duplicates. Two-sided Student’s *t*-test. **b** BMDCs were co-cultured with WT EPEC (MOI of 5) for 2 h, followed by a 2 h gentamicin treatment. Intracellular bacteria were enumerated and CFU at this time-point (4 hpi) were considered 100%. In parallel, gentamicin (50 µg/ml) treatment was allowed to continue for an additional 3 h (total 7 hpi) before enumeration. Data represent mean percentage CFU ± SEM of three independent experiments in duplicates at 4 and 7 hpi. Two-sided Student’s *t*-test. **c** Transduced THP-1 cells were co-cultured with *S. flexneri* M90T for 30 min (MOI of 1) prior to gentamicin treatment for 30 or 90 min. Intracellular live bacteria were enumerated at 1 or 2 hpi. Data represent mean ± SEM of three independent experiments in duplicates. Two-sided Student’s *t*-test. **d** Untransduced WT or WASp shRNA THP-1 cells were co-cultured with *S. flexneri* M90T for 1 or 2 h (MOI of 1). After co-culture, IL-1β levels were quantified by ELISA. Data presented are mean ± SEM of three independent experiments performed in duplicates. Two-sided Student’s *t*-test. **e** THP-1 cells were transduced with mCherry or mCherry-WASp for 3 days. Sorted cells were differentiated and co-cultured with *S. flexneri* M90T for 30 min (MOI of 1) prior to gentamicin treatment for 30 or 90 min. Viable bacteria were enumerated at 1 or 2 hpi. Data represent fold difference in bacterial replication (± SEM) relative to WT cells. Data presented are from three independent experiments in triplicates. Two-sided Student’s *t*-test. **f** Transduced THP-1 cells were co-cultured with *S. flexneri* M90T for 30 min (MOI of 1) prior to 90 min treatment with gentamicin. IL-1β levels were quantified by ELISA. Data are presented as fold change (± SEM) compared to levels obtained from infected WT cells. Data presented are from three independent experiments in duplicates. Two-sided Student’s *t*-test. *p < 0.05; **p < 0.01

EPECs and BMDCs were co-cultured (2 and 3 h) and subsequently treated with gentamicin for 2 h to kill adherent extracellular bacteria^36^. A trend for increase in intracellular bacteria in WAS KO compared to WT BMDCs was observed at 4 hpi (2 h co-culture + 2 h gentamicin treatment), and this difference gained statistical significance at 5 hpi (Fig. 3a). The lack of statistical difference in intracellular bacteria at 4 hpi suggested that similar bacterial uptake had been achieved in both cell types at this time-point; however, the marked increase in intracellular bacteria observed in WAS KO BMDCs at 5 hpi suggested that BMDCs exhibit impaired bacterial handling in the absence of WASp.

The differential increase in intracellular bacteria in infected WAS KO BMDCs could be due to (a) increased bacterial survival and/or (b) decreased bacterial killing. To identify the mechanism(s) responsible, BMDCs were infected for 2 h, followed by a 2 h gentamicin treatment; intracellular bacteria at this time-point (4 hpi) were enumerated and designated as 100%. In parallel experiments, the gentamicin treatment was allowed to proceed for an additional 3 h (7 hpi) prior to bacterial enumeration. We observed a reduction in colony-forming units (CFUs) in infected WT BMDCs between 4 and 7 hpi, suggesting restriction of intracellular bacterial replication (Fig. 3b). In contrast, a significant increase in bacterial load was recorded in infected WAS KO BMDCs. This increase was clearly indicative of active bacterial replication, most likely resulting from abrogation of bacterial clearance.

We next employed a well-studied cytosolic enteropathogen *S. flexneri* to test the role of WASp in modulating bacterial processing. Due to marked BMDC cell death in response to *S. flexneri*, phorbol 12-myristate 13-acetate (PMA)-differentiated WT and WASp short hairpin RNA (shRNA)-knockdown THP-1 cells were employed (Supplementary Fig. 3A). THP-1 cells were infected with *S*. *flexneri* for 30 min prior to gentamicin treatment for 30 or 90 min (1 and 2 hpi, respectively) before cell lysis and bacterial enumeration (Fig. 3c). The infected cells showed minimal variation in intracellular bacterial numbers at 1 hpi; however, a significant difference between the two cell types became apparent at 2 hpi (Fig. 3c). As noted for EPEC-BMDC interactions (Fig. 3a), WASp deficiency had minimal apparent impact on early *S. flexneri* uptake by THP-1 cells (Fig. 3c). Taken together, these data suggest that WASp does not play a major role in the uptake of EPEC and *S. flexneri*, instead WASp appears to be critical in the homeostatic regulation of intracellular bacterial replication and processing. A statistically significant increase in IL-1β paralleled the bacterial counts in WASp shRNA-knockdown cells (Fig. 3d). To rescue the phenotype, similar co-culture studies were performed with mCherry vector transduced WT, WASp shRNA knockdown (controls) and mCherry-WASp transduced into WASp shRNA-knockdown cells (Supplementary Fig. 3B, C). Complementation with WASp restrained both the number of intracellular bacteria and the level of IL-1β secretion to that of vector-alone-transduced WASp shRNA-knockdown cells (Fig. 3e, f).

### Reduced actin recruitment and septin cage formation in WAS

**Fig. 4:**
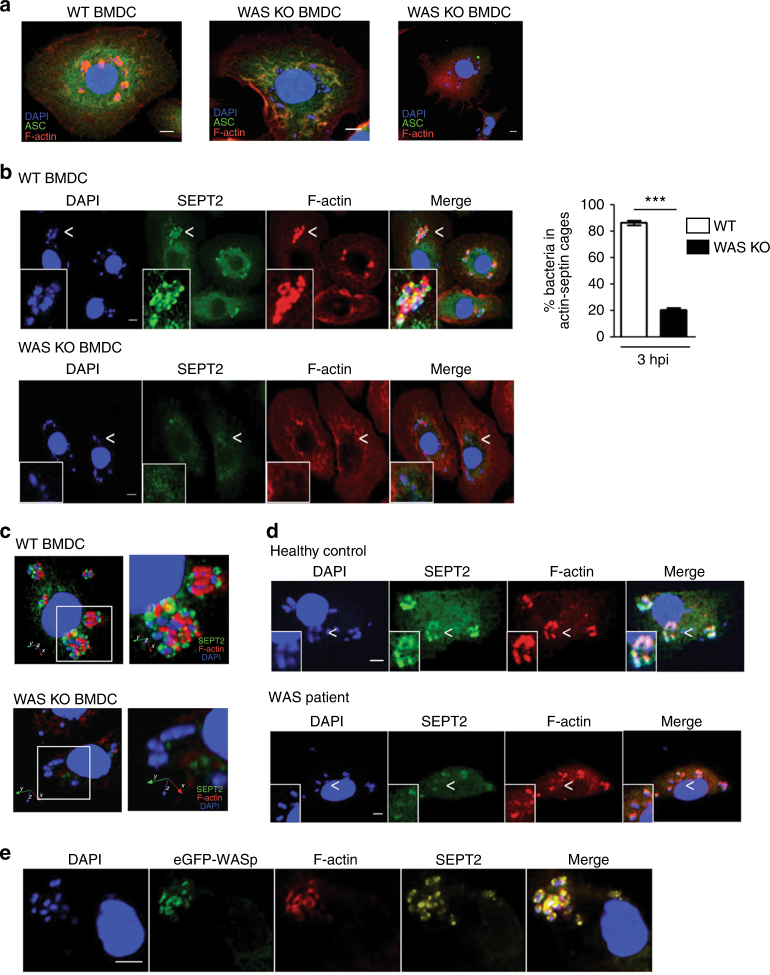
Reduced actin recruitment and septin cage formation in WAS. **a** WT (left) or WAS KO BMDCs (middle and right) were co-cultured with WT EPEC (MOI of 5). At 3 h after co-culture, cells were fixed and stained for bacteria (DAPI (4′,6-diamidino-2-phenylindole)), ASC (green) and F-actin (red). Images were taken by confocal microscopy (×63 magnification). Scale bar = 5 µm. **b** WT (left upper panel) or WAS KO (left lower panel) BMDCs were co-cultured with WT EPEC at an MOI of 5. At 3 h after co-culture, cells were fixed and stained for bacteria (DAPI), SEPT2 (green) and F-actin (red). Images were taken at ×63 magnification. Scale bar = 5 µm. Percentage of bacteria enclosed by F-actin and SEPT2 were enumerated (right). Quantification was performed on at least 1,000 bacteria from two independent experiments. Data are presented as mean ± SEM. Two-sided Student’s *t*-test. ***p < 0.001. **c** Three-dimensional reconstruction of septin cages entrapping EPEC in WT (upper panel) and WAS KO BMDCs (lower panel) is shown (×63 magnification). Scale bar = 5 µm. See also Supplementary Movies 1 and 2. **d** Monocyte-derived macrophages from a healthy control (upper panel) and a WAS patient (lower panel) were co-cultured with WT EPEC (MOI of 5). At 3 h after co-culture fixed cells were stained for bacteria (DAPI), SEPT2 (green) and F-actin (red). Images were taken at ×63 magnification. Scale bar = 5 µm. **e** WAS KO BMDCs were transduced with lentiviral vector expressing eGFP fused to human WASp. Transduced cells were co-cultured with WT EPEC (MOI of 5). At 3 h after co-culture, infected BMDCs were fixed and stained for SEPT2 (red) and F-actin (yellow). WASp-GFP (green), SEPT2 and F-actin formed ring-like structures around bacteria. Images were taken at ×63 magnification. Scale bar = 5 µm

EPEC forms attaching-effacing lesions upon contact with the intestinal epithelium, and the EPEC effector protein EspT is known to activate epithelial Rho GTPases (Rac1 and Cdc42), leading to membrane ruffling and bacterial invasion^34^. The internalized EPEC associate with filamentous actin structures and are capable of intracellular replication^32, 33^. In contrast to the epithelium, how EPEC engages with the innate immune system remains largely unknown. We investigated the role of WASp in EPEC-mediated BMDC actin rearrangements by confocal microscopy. After infection, we observed intense actin recruitment around EPEC in WT cells at 3 hpi (Fig. 4a, left). Strikingly, the majority of bacteria in WAS KO BMDCs showed minimal actin recruitment (Fig. 4a, middle), suggesting a major defect in actin dynamics in the absence of WASp. ASC speck formation accompanied the presence of free bacteria in WAS KO BMDCs (Fig. 4a, right).

Next, we tested if the actin-based structures surrounding bacteria in WT BMDCs were associated with septins, a GTP-binding cytoskeletal protein family known to form cage-like structures and target intracytosolic actin polymerizing bacteria to autophagy^20, 37^. The assembly of septin cages is critically dependent on actin polymerization, as depletion of N-WASP or treatment with cytochalasin D inhibits the formation of septin cages^20^. In infected WT BMDCs, actin polymerizing EPECs were surrounded by SEPT2 (Fig. 4b, upper panel), a septin previously shown to be crucial for septin assembly and function^20, 37–39^. We observed intimate association between EPEC, SEPT2 and F-actin (Fig. 4b, left upper panel), and three-dimensional reconstruction highlighted septin cage-like structures surrounding the bacterium (Fig. 4c and Supplementary Movies 1, 2). In WAS KO-infected BMDCs, the majority of bacteria were not recognized by SEPT2; occasionally, actin or SEPT2 did co-localize with bacteria, but this association was infrequent (Fig. 4b (left lower panel) and Fig. 4c). Consistently, the percentage of EPEC co-localizing with actin and SEPT2 was significantly reduced in WAS KO compared to WT infected cells (20.4 ± 12.7% vs. 86.1 ± 13.8%, respectively, *p* < 0.001, Fig. 4b, right panel). To ensure that the lack of SEPT2 co-localization with bacteria in WAS KO BMDCs was not due to difference in SEPT2 expression, SEPT2 expression in WT and WAS KO BMDCs was investigated. The absence of WASp had no obvious impact on SEPT2 expression (Supplementary Fig. 4).

We next investigated EPEC/SEPT2/F-actin association in monocyte-derived macrophages from a healthy control and from a WAS patient (Fig. 4d). In the healthy control, clear association between the bacterium, SEPT2 and F-actin was observed (Fig. 4d, upper panel). Interestingly, in WAS macrophages infected with EPEC only modest recruitment of SEPT2 and F-actin to individual bacterium was observed, and recruitment of both SEPT2 and F-actin to the same bacterium was rarely seen (Fig. 4d, lower panel). These observations assign a novel role for WASp in bacterial entrapment by septin cage-like structures.

To confirm a role for WASp in septin cage formation, eGFP-WASp-transduced WAS KO BMDCs were employed. In infected cells, eGFP-WASp co-localized with actin in close proximity to SEPT2, and EPEC was entrapped in cage-like structures formed by eGFP-WASp, F-actin and SEPT2 (Fig. 4e). The restoration of F-actin and SEPT2 recruitment in the presence of WASp provides unequivocal evidence that WASp is necessary for successful entrapment of intracellular bacteria in septin cages in macrophages and DCs.

### WASp deficiency leads to impaired bacterial autophagy

**Fig. 5:**
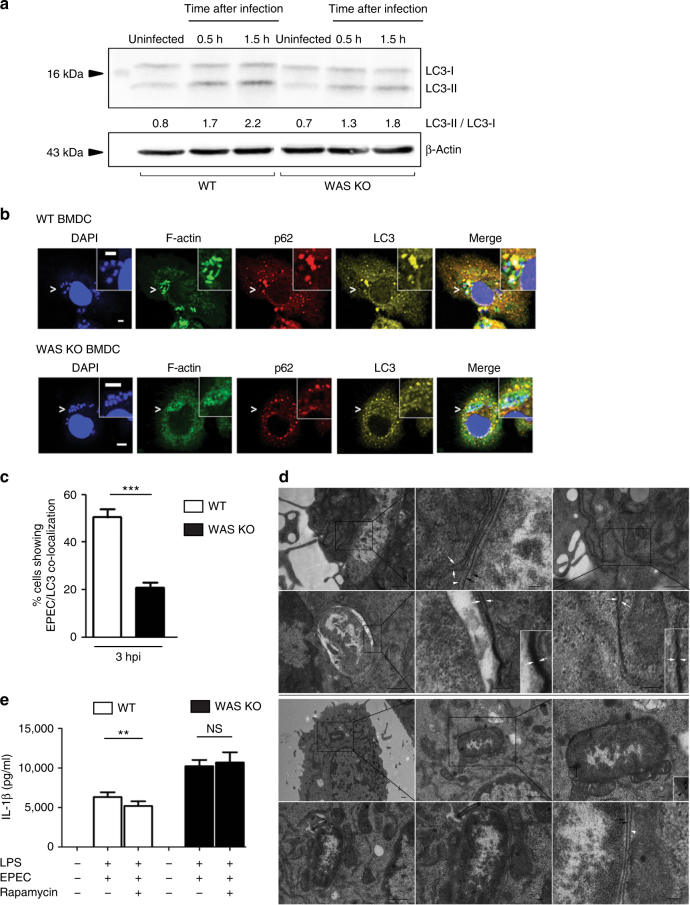
WASp deficiency leads to impaired bacterial autophagy. **a** WT or WAS KO BMDCs were co-cultured with WT EPEC (MOI of 5) for 0.5 or 1.5 h. After co-culture, uninfected control and infected cell lysates were immunoblotted for LC3-I and LC3-II expression. β-Actin was used as a loading control. A representative of three experiments is shown. **b** WT or WAS KO BMDCs were co-cultured with WT EPEC (MOI of 5) for 3 h. Infected cells were fixed and stained for bacteria (DAPI), F-actin (green), p62 (red) and LC3 (yellow). Co-localization of p62 and LC3 to DAPI-bacteria compartmentalized within an actin-based structure in WT (upper panel) and not in WAS-KO (lower panel) BMDCs. Images were taken at ×63 magnification. Scale bar = 5 µm. **c** Percentage of BMDCs showing close association between WT EPEC (MOI of 5) and LC3 puncta 3 h after co-culture. Quantification was performed on at least 500 BMDCs. Data presented are from two independent experiments done in triplicate. Data are presented as mean ± SEM. Two-sided Student’s *t*-test. **d** WT or WAS KO BMDCs were co-cultured with WT EPEC (MOI of 5) for 3 h, followed by gentamicin treatment for 2 h. After infection, cells were fixed and processed for TEM, followed by image acquisition. The black boxed region of the TEM image is enlarged and shown. The scale bar represents 500 nm (first row, left and right; second row, left; third row, left and middle; fourth row, left) and 100 nm (first row, middle; second row, middle and right; third row, right; and fourth row, middle and right). **e** BMDCs were treated with rapamycin for 2 h, followed by EPEC infection (MOI of 5) for 3 h. Rapamycin was maintained throughout the period of infection. Culture supernatants were collected for IL-1β quantification by ELISA. Data are presented as mean protein concentration ± SEM from three independent experiments in duplicate. Two-sided Student’s *t*-test. **p = 0.01; ***p < 0.001

Septin cage-entrapped bacteria are recognized by ubiquitin-binding adaptor proteins such as p62 and targeted to autophagy, a process that culminates in the conversion of LC3-I to its lipidated form, LC3-II^37–39^. We compared the kinetics of autophagy in EPEC-infected WT and WAS KO BMDCs by studying LC3-I/LC3-II expression during infection. In uninfected cells, basal autophagy was comparable between the two cell types. Upon co-culture, increased LC3 conversion was noted in infected WT BMDCs, and this was observed as early as 30 min after infection (Fig. 5a). In contrast, infected WAS KO BMDCs showed slower kinetics and degree of conversion (Fig. 5a and Supplementary Fig. 5A).

As the rate of LC3 conversion differed modestly between infected WT vs. WAS KO BMDCs when assessed by western blotting, we sought confirmation by enumeration of LC3 puncta and their spatial association with p62-associated EPEC by confocal microscopy. Few LC3 puncta were observed in uninfected WT and WAS KO BMDCs, and this was also observed in eGFP-transduced WT and WAS KO BMDCs (Supplementary Fig. 5B). In infected WT BMDCs, F-actin recruitment to EPEC was observed; in addition, p62 and LC3 co-localized with EPEC and actin (Fig. 5b, upper panel). In contrast, in infected WAS KO BMDCs, F-actin expression remained diffused and the intensity of p62 and LC3 co-localization with EPEC was poor (Fig. 5b, lower panel). Overall, the percentage of EPEC that co-localized with LC3 was significantly lower in WAS KO compared to WT cells (Fig. 5c).

To confirm a potential role for WASp in regulating canonical autophagy, transmission electron microscopy (TEM) was performed on EPEC-infected WT and WAS KO BMDCs (Fig. 5d). Phagocytosed bacteria in infected WT (first and second rows) and WAS KO (third and fourth rows) BMDCs were observed. The inner and outer bacterial membranes (black arrows) of engulfed bacteria can be seen in WT (first row, middle and second row, middle) and in WAS KO BMDCs (third row, right and fourth row, right). In WT BMDCs, rough endoplasmic reticulum (RER) membranes consistently associated with phagocytosed bacteria (Fig. 5d, first panel, left); this association is highlighted in Fig. 5d (first panel, middle). Evidence of transition of bacterial-associated RER membranes to classic autophagy (the presence of osmophilic di-membranes) was clearly observed in Fig. 5d (second panel, left and middle). In addition to bacterial autophagy, we were able to record early stages of cellular autophagy (omegasome formation and presence of double ER membranes) as shown in Fig. 5d (first panel, right) and Fig. 5d (second panel, right) (low and high magnification). We next examined phagocytosed bacteria in infected WAS KO BMDCs (Fig. 5d, third and fourth panel). In this case, although the bacterial membranes were easily identified, no association with rough endoplasmic reticulum di-membranes was recorded, and instead the presence of a single host membrane was observed (Fig. 5d, third panel, right). In all phagocytosed bacteria studied, canonical autophagosome formation was not detectable in the absence of WASp. These findings suggest a major defect in autophagosome formation in the absence of WASp.

To understand if autophagocytic defects were restricted to bacterial challenge, BMDCs were treated with rapamycin and bafilomycin (well-described modulators of autophagic activity). Treated cells showed attenuated LC3 conversion, suggesting an intrinsic deficiency in autophagy in the absence of WASp (Supplementary Fig. 5C). Consistent with this, LC3 conversion in rapamycin-treated WAS KO THP-1 cells was also attenuated (Supplementary Fig. 5D, E). Collectively, these data indicate that WASp is involved in bacterially and chemically induced autophagy.

Autophagy is viewed to down-regulate the inflammasome^13–15^, so pre-treatment with rapamycin prior to EPEC infection should cause reduced inflammasome activation compared to infection alone. This is indeed the case in infected WT BMDCs (Fig. 5e). However, this effect is not observed in infected WAS KO BMDCs, raising the paradigm that by regulating bacterial autophagy an intact actin cytoskeleton plays an important role in preventing hyperactivation of the inflammasome.

### Type I interferons suppress inflammasome activation in WAS

**Fig. 6:**
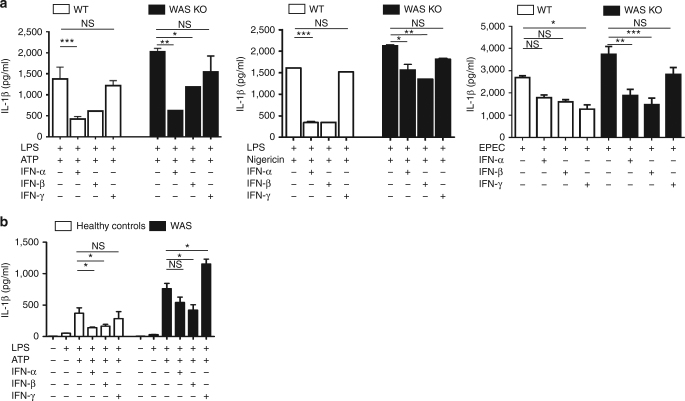
Type I interferons suppress inflammasome activation in WAS. **a** WT or WAS KO BMDCs were stimulated with type-1 IFNs or IFN-γ (500 U/ml) for 16 h. Cells were then primed with LPS (100 ng/ml) for 3 h, followed by stimulation with mM ATP for 30 min (left); 10 μM nigericin for 1 h (middle) or WT EPEC (MOI of 5, right) for 3 h. IL-1β protein levels were quantified by ELISA. Cytokine levels from three independent experiments performed in duplicate are shown as mean protein concentration ± SEM. Two-sided Student’s *t*-test. **b** PBMCs from healthy controls (*n* = 3) or WAS patients (*n* = 3) were stimulated with IFN-α, IFN-β or IFN-γ for 16 h, followed by LPS (100 ng/ml) for 3 h and then followed by ATP (3 mM) stimulation for 30 min. Culture supernatants were subjected to IL-1β quantification by ELISA. Results are presented as mean protein concentration ± SEM from three independent experiments performed in duplicate. Two-sided Student’s *t*-test. *p < 0.05; **p < 0.01; ***p < 0.001; NS = not significant

Interferon-α (IFN-α) and IFN-β (collectively referred as type I IFNs) are known to exert inhibitory effects on inflammasome activation^40, 41^. We hypothesized that heightened inflammasome activation in WAS might be ameliorated by exogenous IFNs. To test the inhibitory potential of IFNs on inflammasome activation, WT and WAS KO BMDCs were pre-incubated with IFN-α or IFN-β for 16 h before subjecting to standard LPS priming and ATP/nigericin stimulation. IFN-γ, a potent bactericidal cytokine, was included for comparison (Fig. 6a, left). As noted above (Fig. 1b), WAS KO BMDCs secreted significantly greater IL-1β than WT cells upon exposure to ATP. IFN-α and IFN-β were equipotent in mediating significant inhibition of cytokine secretion in WT cells, while the effect of IFN-γ was not significant (Fig. 6a, left). Both type I IFNs exerted a statistically significant impact on inflammasome activity in WAS KO BMDCs, suggesting that pathways involved in this inhibition were largely intact in the absence of WASp. The inhibitory effect was also observed in response to nigericin (Fig. 6a, middle).

Next, we investigated the impact of IFNs on inflammasome activation in response to infectious stimuli. In EPEC-BMDC co-cultures, an infection model that inadvertently added greater complexity to the autophagy-inflammasome crosstalk compared to the sterile LPS/ATP system employed above, the effect of IFNs was variable. Not surprisingly, in this model IFN-γ mediated significant impact on IL-1β secretion in WT BMDCs (Fig. 6a, right). This was most likely due to the potent bactericidal effect of IFN-γ. When tested, this was indeed the case as IFN-γ pre-treatment led to a significant decrease in the number of intracellular bacteria whilst IFN-α and IFN-β pre-treatment had minimal impact on the number of intracellular bacteria (Supplementary Fig. 6).

Due to limited patient sample availability, similar experiments were restricted and performed only on peripheral blood mononuclear cells (PBMCs) from healthy controls and WAS (Fig. 6b). Pre-treatment with type I IFNs caused significant inhibition in LPS/ATP-mediated inflammasome activity in healthy controls, and the impact of IFN-γ was minimal. Type I IFNs also showed a trend for inflammasome inhibition in WAS PBMCs, an inhibition that gained significance in response to IFN-β. For reasons unclear, IFN-γ pre-treatment led to an increase in IL-1β in WAS PBMCs. Although the effect of IFNs varied, this series of experiments highlights a potential therapeutic role for type I IFNs in resolving dysregulated inflammasome function in WAS.

## Discussion

WAS patients are prone to develop complex autoinflammatory and autoimmune manifestations, largely due to intrinsic and cooperative dysregulation of the immune system^42–47^. The contributory factors to exaggerated inflammation have not been explored, although increasing evidence from various disease models suggests that there are important mechanistic links with sustained autoimmunity. To our knowledge, the present study is the first to report a link between the actin cytoskeleton and inflammasome activity in WAS, and the first to demonstrate a crucial role for WASp in autophagocytic handling of bacteria. We explored how WASp deficiency, as a model of cytoskeletal dysfunction, may impact on inflammasome activation after chemical and bacterial challenge. WASp-deficient monocytes from patients with classical WAS (Fig. 1a) and WAS KO murine BMDCs (Fig. 1b–e and Supplementary Figs 1 and 2) exhibit increased caspase-1 activity upon NLRP3 activation by ATP and nigericin. Using ex vivo models of EPEC and *S. flexneri* infection, a significant increase in IL-1β secretion and pyroptosis occurred in response to both infectious agents in BMDCs and THP-1 cells in the absence of WASp (Figs 2 and 3). Collectively, our observations of heightened ASC speck formation, IL-1β secretion and cell death highlights the importance of WASp-mediated actin assembly in regulating inflammasome function during infection and inflammation.

Our findings suggest for the first time that WASp-mediated actin polymerization is essential for recruiting septins and targeting bacteria to autophagy in innate immune cells. Previous studies have shown that actin polymerization and N-WASP are required for *Shigella*-septin cages, which restrict intracellular bacterial proliferation in HeLa cells^19–21, 26, 29^. Bacterial mutants unable to polymerize actin, or disruption of F-actin by cytochalasin D dramatically reduced the number of *Shigella*-septin cages^19, 38^. Septin assembly is implicated in the recruitment of autophagy components, p62 and LC3 do colocalize with *Shigella*-septin cages and the depletion of septins significantly impairs autophagic activity^19, 38^. Recent work has shown that septin cages restrict the cytosolic proliferation of *S. flexneri* ^21^. Herein, we report that SEPT2 and F-actin are recruited to EPEC and form similar cage-like structures in murine and human innate immune cells. Septin cage formation was significantly diminished in WAS KO cells and could be reconstituted by re-introducing WASp. The co-localization of WASp with F-actin and SEPT2 provides strong evidence that WASp actively participates in the structural assembly of the entrapping cages.

A hallmark of canonical autophagy is the sequestration of the cargo (e.g. bacteria) into a double-membrane structure, the autophagosome^13, 15, 16^. TEM allowed us to identify a major defect in canonical bacterial autophagy in the absence of WASp, as unlike in WT BMDCs, no association of RER di-membranes with phagocytosed bacteria was recorded. Collectively, these observations can be used to explain the persistence of internalized bacteria in the absence of WASp. We propose that the cooperative (or linked) effect of defective septin cage formation and the failure of canonical autophagosome formation promotes bacterial survival and replication, processes detrimental to host cell homeostasis.

WHAMM (WAS protein homolog associated with actin, Golgi membranes and microtubules), JMY (junction-mediating and regulating protein) and WASH (Wiskott–Aldrich syndrome protein and SCAR homologue), members of the class I actin nucleation promoting family^48^ have been previously implicated in autophagy^49, 50^. WHAMM is involved in autophagosome biogenesis via the Arp2/3 complex^51^; JMY promotes autophagy by nucleating actin in the autophagosome, leading to enhanced cell survival^27^ while WASH is required for cargo delivery to nascent autophagosomes^52^. Herein, we propose that WASp, the founding member of this actin nucleation-promoting family is also involved in homeostatic regulation of autophagy as no canonical bacterial autophagy was observed in its absence. The lack of double-membrane autophagosomes and the presence of single membrane-entrapped bacteria in infected WAS KO BMDCs raises interesting new questions. LC3-associated phagocytosis (LAP) occurs at the cusp of phagocytosis and autophagy. Further studies are therefore required to determine to what extent the single-membrane compartments observed in the absence of WASp represent LAP, and to delineate the contribution of WASp to both canonical and non-canonical autophagy pathways.

Our findings provide a direct link between actin assembly, autophagy and the inflammasome pathways. One may speculate that as autophagosome formation is dysfunctional in WAS innate immune cells, these cells are primed for hyperinflammation whether as a result of microbial challenge or through ‘sterile’ cellular stress. This may partly explain the enhanced inflammasome activation observed after chemical stimuli, as autophagic regulation is intrinsically compromised.

The activation of the inflammasome is tightly controlled by various cell-intrinsic and cell-extrinsic mechanisms to prevent tissue damage. For example, type I IFNs restrain IL-1β production by (a) diminishing the pool of intracellular pro-IL-1β and caspase-1-dependent IL-1β maturation, and/or (b) inducing IL-10, which signals via signal transducer and activator of transcription 3 (STAT3) to reduce the abundance of pro-IL-1β^40^. Additionally, type I IFNs suppress caspase-1 processing by a STAT-1-dependent mechanism. It has previously been reported that WAS KO conventional, plasmacytoid and splenic DCs show impaired type I IFN production in response to TLR ligation^53^. Interestingly, type I IFNs are able to inhibit IL-1β secretion even in the absence of WASp, raising the possibility that type I IFN therapy is useful for pathological states related to overt inflammasome activation in WAS patients, as well as for enhancing protective immunity against pathogens to which patients are particularly susceptible in natura, such as herpes viruses.

Our novel findings support the hypothesis that WASp provides an important mechanistic link between actin-mediated autophagy and inflammasome activity in innate immune cells. Interestingly, PAPA syndrome (pyogenic sterile arthritis, pyoderma gangrenosum and acne) caused by mutations in the cytoskeletal adapter PSTPIP1 (which binds to WASp) shares cytoskeletal features with WAS and is also associated with overt IL-1β production^54^. In a recent murine study, an inactivating mutation of the actin-depolymerizing co-factor Wdr1 resulted in systemic autoinflammation mediated by caspase-1, pyrin and IL-18^25^. In humans, *WDR1* dysfunction is a cause of an autoinflammatory syndrome characterized by periodic fever, immunodeficiency and thrombocytopenia^6^. Patient monocytes showed increased caspase-1 cleavage, but in contrast to our findings with WAS monocytes, WDR1 monocytes secreted high levels of IL-18 but not IL-1β. In addition, *WDR1*-mutated DCs contained abnormal WDR1 aggregates. Thus, genetic defects in *WAS* and *WDR1* are clear examples of human syndromic primary immunodeficiencies with excessive inflammasome activation linked to dysregulation of the actin cytoskeleton, albeit with different pathogenetic mechanisms.

To conclude, this study provides evidence for an important link between the actin cytoskeleton, autophagy and inflammasome activation, processes crucial for maintaining immune homeostasis, with direct relevance to human disease. We propose that an intrinsically primed inflammasome might act as a driver for chronic inflammation and autoimmunity in WAS. These results may inspire a rational target for therapeutic intervention where inflammatory complications are prominent.

## Methods

### Patients and human cell lines

Peripheral blood was obtained from healthy individuals, three patients with classical WAS and two patients with X-linked thrombocytopenia with known hypomorphic missense mutations (p.L39R and p.T35M) in the *WAS* gene. Informed written consent was obtained in accordance with the Declaration of Helsinki and ethical approval from the Great Ormond Street Hospital for Children NHS Foundation Trust and the Institute of Child Health Research Ethics (08/H0713/87), and The University of Hong Kong/Hospital Authority Hong Kong West Cluster (HKU/HA HKW) Institutional Review Board. PBMCs were collected using Ficoll-Hypaque gradient separation, and monocytes were isolated by MACS CD14^+^ selection according to the manufacturer’s instructions (Miltenyi Biotech, Cat. #131-050-201). Monocyte-derived macrophages were obtained by culturing CD14^+^ monocytes with recombinant human macrophage colony-stimulating factor (Peprotech, UK) at 20 ng/ml for 5 days.

THP-1 cells from American Type Culture Collection (ATCC #TIB-202) were maintained in RPMI-1640 medium containing 10% foetal calf serum and antibiotics. Regular checks for mycoplasma contamination were performed. WAS KO THP-1 cells were generated as follows: 2 × 10^5^ THP-1 cells were nucleofected (Amaxa 4D Nucleofector; program FF-100; Lonza) with 3 μg Cas9 protein complexed with 1 μg of guide RNA targeting the first exon of the *WAS* gene. After 48 h, cells were FACs sorted (Aria III FCF; Beckton Dickinson) into single wells and expanded to monoclonal cell lines. Clones were screened for the presence of indels (insertions or deletions) at the guide RNA target site by PCR amplification and Sanger sequencing. KO in selected edited clones was assessed and confirmed by western blotting. Cells were differentiated into macrophages by treatment with 10 ng/ml PMA (Sigma-Aldrich) for 24 h, followed by rest for 24 h before co-culture.

### Lentiviral vectors

To generate WASp-knockdown THP-1, cells were transduced with a vesicular stomatitis virus G-pseudotyped lentivector with the W7 shRNA construct targeting WASP, as previously described^55^. The lentivector was prepared in the pLN-SEW-TH lentiviral backbone (Supplementary Fig. 3A). The transduced THP-1 cells were subcloned to obtain a clonal population that expressed <10% WASp compared with untransduced cells.

Lentiviral vectors expressing mCherry and mCherry fused to human WASp were prepared in the Sffv-mCherry-WASp-WPRE lentiviral backbones. The plasmid map is shown in Supplementary Fig. 3B. Undifferentiated scrambled control (GFP^+^) THP-1 cells were transduced with mCherry lentivector (multiplicity of infection (MOI) of 10), and WASp shRNA-knockdown (GFP^+^) THP-1 cells were transduced with either mCherry or mCherry-WASp lentivector (MOI of 10), resulting in transduction of 99% and 39% of population, respectively, after 3 days. Post-transduction GFP-mCherry-positive cells were FACs sorted (Supplementary Fig. 3C).

### Mice

C57BL/6 WT (Charles River), WAS KO mice (originally supplied by T. Strom, Memphis, TN, USA and Jackson laboratories) were housed in specific pathogen-free conditions. Both male and female mice were used at 6–18 weeks of age. All animal work was approved by the Institutional Research Ethics Committee (Institute of Child Health, University College London, UK) and performed under UK government Home Office Project License Number 70/7024. For BMDCs, bone marrow was flushed from femurs and tibia and plated in RPMI medium containing 10% foetal calf serum, 100 U/ml of penicillin and 100 μg/ml of streptomycin in the presence of 20 ng/ml of murine GM-CSF (Peprotech) for 6 days.

### Reagents

LPS from *E. coli* 026:B6 (Sigma-Aldrich) and ATP (Invivogen) were used at concentration of 50 ng/ml and 3 mM for human and 100 ng/ml and 5 mM for murine co-culture studies, respectively. Nigericin (Invivogen) was used at a concentration of 10 µM. To evaluate autophagy response, WT and WAS KO BMDCs or WT and WAS KO THP-1 cells were stimulated with rapamycin (50 nM; Calbiochem) and bafilomycin (160 nM; Thermo Fisher Scientific) alone or in combination. All murine IFNs were from Millipore and human cytokines from Peprotech. BMDCs were stimulated with 500 U/ml IFN for 16 h prior to LPS priming (100 ng/ml) for 3 h, followed by ATP (5 mM) for 30 min.

### Bacteria

WT E2348/69 EPEC was a kind gift from Professor G. Frankel (Imperial College, London, UK)^56^. For EPEC culture, 1 ml of unsupplemented Dulbecco’s modified Eagle’s medium media (Gibco, Invitrogen) was inoculated with 25 μl of an overnight bacterial culture (in Lysogeny broth (LB)) and incubated for 2 h at 5% CO_2_ at 37 °C. This protocol is known to enhance the expression of bacterial virulence genes^36^. Bacterial numbers were enumerated as absorbance at 600 nm. *S. flexneri* strain M90T was streaked onto Tryptic soy Congo red agar plates. The starter exponential phase culture was prepared by inoculating fresh LB media with overnight culture (1:80 ratio). Bacteria were enumerated whenoptical density (600 nm) reached between 0.5 and 0.6.

### Gentamicin protection assay

A total of 2 × 10^5^ BMDCs were infected with EPEC at an MOI of 5. Co-cultures were spun at 1,200 r.p.m. for 5 min to enhance bacteria-BMDC interaction. Media containing bacteria were removed at specified time-points, and washed twice with RPMI containing 100 µg/ml gentamicin. The cells were incubated with the same media for 2 and 3 h, respectively. After incubation, the cells were washed once with sterile phosphate-buffered saline (PBS) before lysis with 0.1% Triton-X (Sigma) in PBS for 5 min. Once lysed, the cellular contents underwent 10-fold serial dilutions. Fifty microlitres of serial diluent was spread onto LB agar plates and incubated for 24 h at 37 °C. Colonies obtained were indicative of the presence of live intracellular bacteria and were enumerated as CFUs.

A total of 0.5 × 10^6^ cells/ml PMA-differentiated THP-1 cells were infected with *S*. *flexneri* at an MOI of 1 (spun at 1,200 r.p.m. for 5 min to promote interactions) for 30 min, followed by 50 μg/ml gentamicin treatment in RPMI media for 30 or 60 min. Infected cells were lysed and CFU enumeration was performed as above.

### Measurement of activated caspase-1, IL-1β and cell death

To detect caspase-1 activation, cells were activated by LPS and nigericin or EPEC infection, followed by labelling with FAM-YVAD-FMK caspase-1 (FAM-FLICA, Immunochemistry, Bloomington, IN, USA) for 30 min. PI (0.5% (v/v)) was added for the last 5 min of incubation to label dead cells. The presence of active caspase-1 and incorporation of PI was analyzed by Cyan flow cytometer (Beckman Coulter) and the FlowJo software. The amount of IL-1β released in the culture supernatant was determined by enzyme-linked immunosorbent assay (ELISA) (R&D Systems, Lille, France). IL-1 receptor expression on BMDC was determined by staining with APC anti-mouse CD121a (IL-1R, JAMA-147; BioLegend) by flow cytometry. Cytotoxicity induced was evaluated by LDH assay using the CytoTox96 LDH-Release Kit (Promega), as described by the manufacturer’s protocols.

### Immunoblotting

After co-culture with LPS/ATP, rapamycin and/or bafilomycin or EPEC (MOI = 5), unstimulated/uninfected and stimulated/infected BMDCs (WT/WAS KO) and THP-1 (WT and WAS KO) were lysed, and whole-cell extracts were quantitated by the Bradford assay (Bio-Rad). Supernatant proteins were precipitated overnight in 80% ice-cold acetone. Equal amounts of cell lysates or supernatants were resolved by sodium dodecyl sulphate-polyacrylamide gel electrophoresis and were transferred to nitrocellulose or polyvinylidene fluoride membrane (GE Healthcare, Buckinghamshire, UK). The membranes then were incubated with polyclonal rabbit anti-mouse IL-1β (1:500, H153; Santa Cruz Biotechnology), monoclonal anti-mouse caspase-1 (1:1,000, Casper-1; Adipogen), polyclonal rabbit anti-mouse LC3 (1:1,000, PM036; MBL International) or polyclonal rabbit anti-mouse SEPT2 (1:1,000, 11397-1-AP; Proteintech), followed by an horseradish peroxidase-conjugated secondary antibody. Membranes were stripped and re-probed for β-actin (1:20,000; Sigma) or glyceraldehyde 3-phosphate dehydrogenase (1:20,000; Thermo Fisher Scientific) as a loading control. The immunoreactive bands were detected using the Amersham ECL Prime Western Blotting Detection Reagent (GE Healthcare).

### Immunofluorescence and confocal microscopy

Cells were seeded onto poly-D-lysine-coated tissue culture slides and allowed to adhere overnight before stimulation by LPS/ATP or EPEC as per the protocol. After stimulation, cells were washed with PBS three times prior to fixation with 4% paraformaldehyde for 20 min, followed by permeabilization with 0.1% Triton X-100 in PBS for 5 min. For LC3 immunostaining, cells were permeabilized with digitonin (100 μg/ml) for 10 min. Non-specific binding was blocked with 5% bovine serum albumin/PBS for 30 min, followed by staining with primary antibodies, which included rabbit anti-ASC (ASC-(N15)-R, sc22514R; Santa Cruz Biotechnology), goat anti-SEPT2 (septin 2 (N-12), sc20408; Santa Cruz Biotechnology), anti-p62 (SQSTM1 (H-290), sc25575; Sana Cruz Biotechnology) and rabbit anti-LC3 (PM036; MBL International). Secondary antibodies included donkey anti-rabbit Alexa Fluor-488, donkey anti-goat Alexa Fluor-488, donkey anti-goat Alexa Fluor-568, mouse anti-rabbit Alexa Fluor-647 (all from Molecular Probes). F-actin was labelled with Alexa Fluor 488- or 647-phalloidin (Molecular Probes). Fluorescent microscopy images were acquired using an inverted Axiobserver Zeiss Z1 microscope, and an LSM 710 confocal microscope. Images were then processed with Fiji (http://fiji.sc/Fiji). 3D images rendering was performed with Icy (http://icy.bioimageanalysis.org), running the 3D VTK plugin on the original file. The 3D axis was kept to display the orientation of the cells, and then a snapshot of the image was taken using the appropriate tool from the same software. The cropped image (inset) was taken on a higher magnification using the same settings from the same picture.

### Transmission electron microscopy

WT and WAS KO BMDCs were infected with EPEC for 3 h, followed by gentamicin treatment as described. After infection, cells were washed and fixed in 0.5% glutaraldehyde in 200 mM sodium cacodylate buffer for 30 min, washed in buffer and secondarily fixed in reduced 1% osmium tetroxide and 1.5% potassium ferricyanide for 60 min. The samples were washed in distilled water and stained overnight at 4 °C in 0.5% magnesium uranyl acetate, washed in distilled water and dehydrated in graded ethanol. The samples were then embedded flat in the dish in Epon resin. Resin-filled stubs were placed on embedded cell monolayers and polymerized. Ultrathin section (typically 50–70 nm) were cut parallel to the dish and examined in an FEI Tecnai electron microscopy with CCD camera image acquisition.

### Statistical analysis

Histograms represent mean, with error bars indicating SEM. Statistical significance was analyzed with GraphPad Prism v.7 software (GraphPad Inc., La Jolla, CA, USA) using two-sided Student’s *t*-test.

### Data availability

The data that support the findings of this study are available from the corresponding author upon request.

## Electronic supplementary material

Supplementary information

Description of Additional Supplementary Files

Supplementary Movie 1

Supplementary Movie 2
