# Supplementary material for: Wiskott-Aldrich syndrome protein regulates autophagy and inflammasome activity in innate immune cells
Source: Nat Commun. 2017 Nov 17;8:1576. doi: 10.1038/s41467-017-01676-0 (PMC5691069; doi:10.1038/s41467-017-01676-0)
Supplement: Supplementary file 1 — Supplementary information [file 41467_2017_1676_MOESM1_ESM.pdf]

Supplementary Figure 1

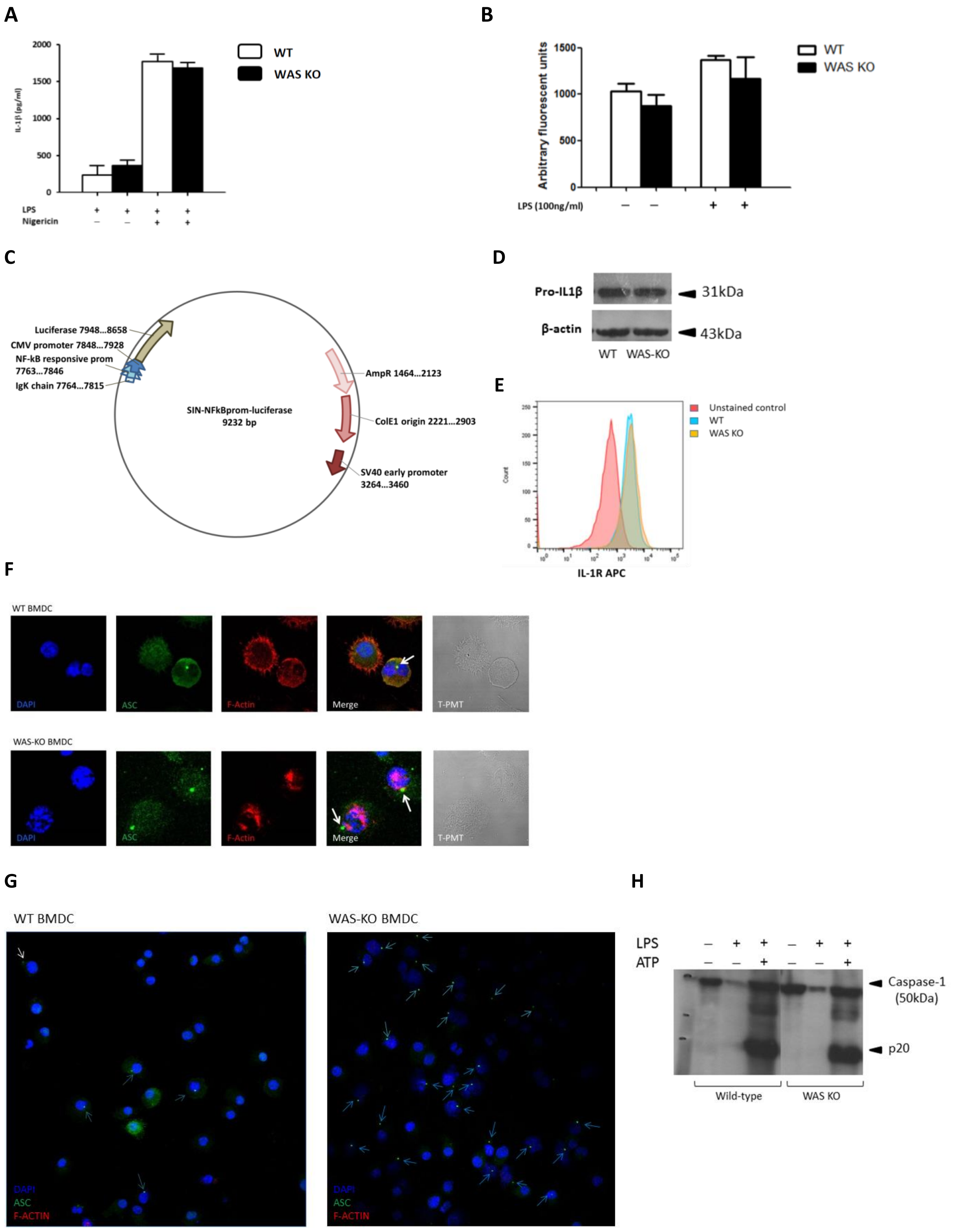

A. CD14+ monocytes from 2 healthy controls and 2 patients with X-linked thrombocytopenia (XLT) were primed with LPS (50 ng/ml) for 3 h followed by nigericin stimulation (10 μM) for 30 min. Post-stimulation, IL-1β protein levels were evaluated in culture supernatants by ELISA. Data presented is mean protein concentration +/- SEM performed in duplicates. Unpaired Student's t-test.

B. BMDCs from WT and WAS-KO mice were transduced with a lentiviral vector containing an NF-kB reporter plasmid (Supplementary Fig. 1C) at an MOI 10. $1x10^5$ transduced BMDCs were stimulated by LPS (100ng/ml) in 96-well plates for 3 hours. Luciferase activity was measured by bioluminescent assay. Data presented is mean protein concentration +/- SEM performed in duplicates. Unpaired Student's t-test.

C. NF-κB inducible lentiviral plasmid. An NF-κB responsive element drives expression of luciferase protein from a minimal cytomegalovirus promoter.

D. BMDCs from WT and WAS KO mice were simulated by LPS (100 ng/ml) for 3 h. Post-stimulation, cell lysates were subjected to SDS PAGE followed by immunoblotting for pro-IL-1β. β-actin was used as loading control.

E. WT and WAS KO BMDC were stained with CD121a antibody (APC) and basal IL-1 receptor expression was evaluated by flow cytometry.

F. WT or WAS KO BMDCs were primed with LPS (100 ng/ml) for 3 h and stimulated with ATP (5 mM) for 10, 20 or 30 min. Cells were fixed and stained for nuclei (blue), ASC (green) and F-actin (red). Images were taken by confocal microscopy (63 × magnification). Representative confocal images of BMDCs containing ASC specks (arrows) after 30 min of ATP (5mM) stimulation at i) high magnification (63×). The presence of ASC speck, rounding of the cell shape and loss of integrity of the actin cytoskeleton of the cell undergoing pyroptosis (pointed with white arrow) is in contrast with a neighbouring cell without ASC speck (upper panel, WT BMDCs). ASC speck formation and morphological changes of pyroptosis are similar in WAS KO BMDCs (lower panel).

G. Quantification of ASC speck at lower magnification (40×) in WT DCs (left panel) and WAS-KO DCs (right panel).

H. WT and WAS KO BMDC were stimulated with LPS (100ng/ml) alone for 3h or in addition with ATP (5mM) for 30min. Post-stimulation, culture supernatants were treated with 80% ice-cold acetone overnight. Resultant protein precipitate was subjected to SDS PAGE followed by immunoblotting for caspase-1 and caspase-1 p20. A representative blot is shown.

Supplementary Figure 2

A.

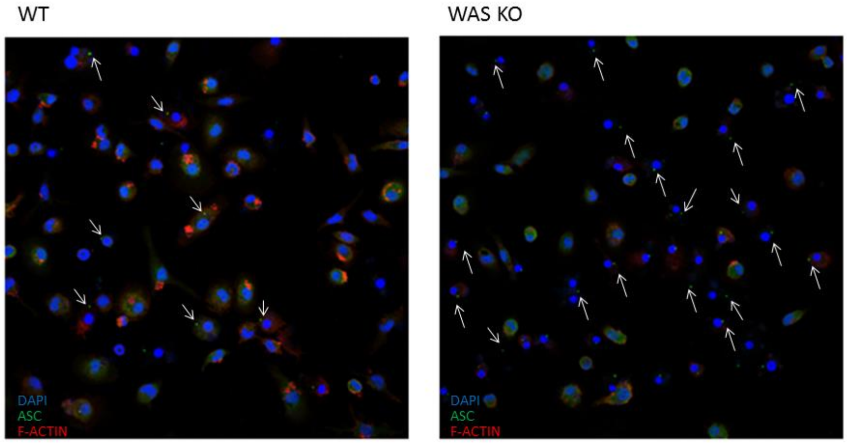

B.

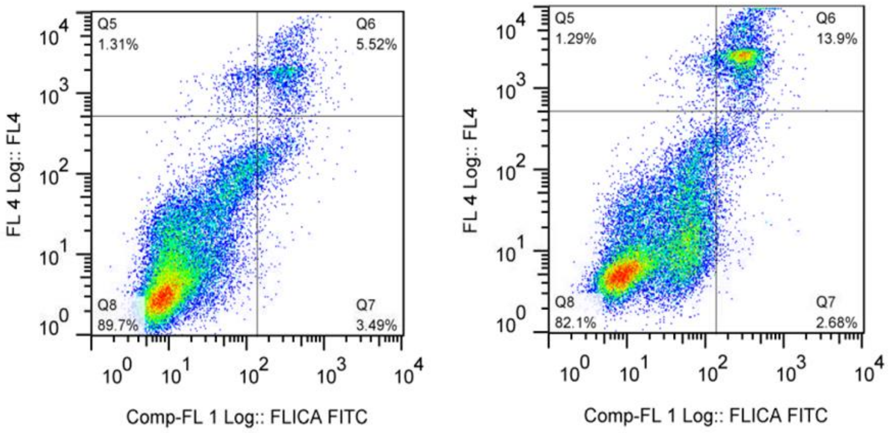

A. WT and WAS KO BMDCs were primed with LPS (100ng/ml) for 3 h followed by co-culture with EPEC (MOI 5) for 3 h. Post-fixation, cells were stained for bacteria (DAPI), ASC (green) and actin phalloidin (red). ASC speck formation was quantified by confocal microscopy at lower magnification (40×) in WT BMDC (left panel) and WAS KO BMDC (right panel). A representative image is shown.

B. WT or WAS KO BMDCs were primed with LPS (100 ng/ml) for 3 h followed by co-culture with WT EPEC (MOI 5) for 3 h. Intracellular active caspase-1 was detected by FAM-YVAD-FMK (FAM-FLICA™) binding, dead cells were stained with propidium iodide (PI). The percentage of FLICA and PI double-positive BMDCs were enumerated by flow cytometry (FLICA collected in FL-1 gate; PI collected in FL-2 gate). A representative flow plot is shown. BMDCs exhibiting pyroptosis were dually stained with FAM-FLICA™ and PI.

Supplementary Figure 3

A

pLN-SEW-TH

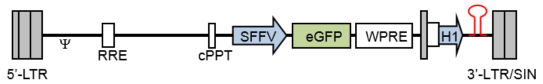

MluI sense strand loop antisense strand stop XhoI

CGCT CCCC NNN...................NNN TTCAAGAGA NNN...................NNN TTTTT GGAA C
A GGGG NNN...................NNN AAGTTCTCT NNN...................NNN AAAAA CCTT GAGC

Name	Target gene	Antisense sequence
W7	WASp	TGAGATGCTTGGACGAAAA

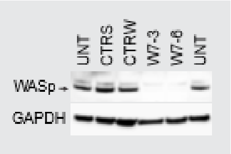

%WASp (±SE)	
CTRS	139±14
CTRW	118±16
W7	28±21
W7-3	7.3±2
W7-6	8.0±1

B

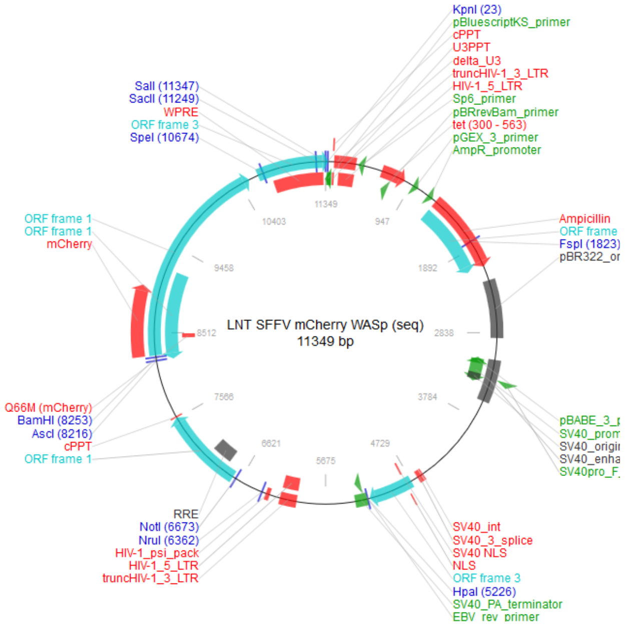

C

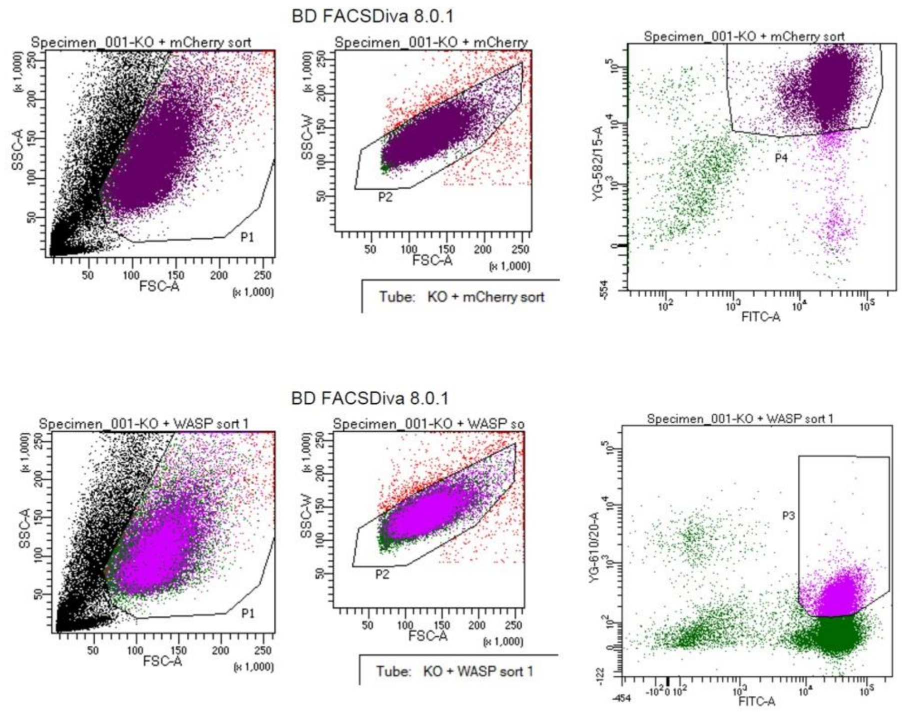

A. Generation of WASp shRNA knockdown THP-1 cells. THP-1 cells were transduced with a VSVg-pseudotyped lentivector with the W7 shRNA construct. Schematic representation of the lentiviral genome of pLN-SEW-TH and WASp shRNA oligonucleotides is shown. Post-transduction, WASp expression was analysed by Western blot. Clone W7-3 was utilized in the current study.

B. Lentiviral vectors expressing mCherry and mCherry fused to human WASp were prepared in the Sffv-mCherry-WASp-WPRE lentiviral backbones. The plasmid map is shown.

C. Undifferentiated scrambled control (GFP+) THP-1 cells were transduced with mCherry lentivector (MOI 10), and WASp shRNA knockdown (GFP+) THP-1 cells were transduced with either mCherry (upper panel) or mCherry-WASp lentivector (lower panel) at MOI 10, resulting in transduction of 99% and 39% of population, respectively, after 3 days. Post-transduction GFP-mCherry-positive cells were FACs-sorted

Supplementary Figure 4

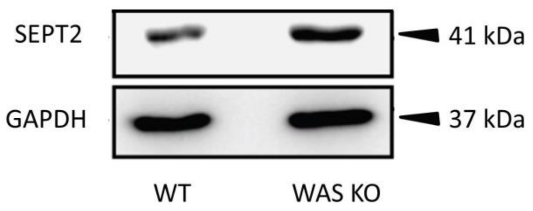

Cell lysates from WT and WAS KO BMDC were subjected to SDS PAGE and immunoblotting for basal SEPT2 expression. GAPDH was used as loading control.

Supplementary Figure 5

A

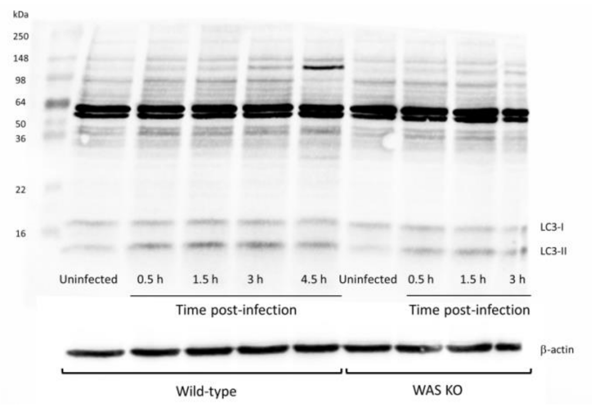

B

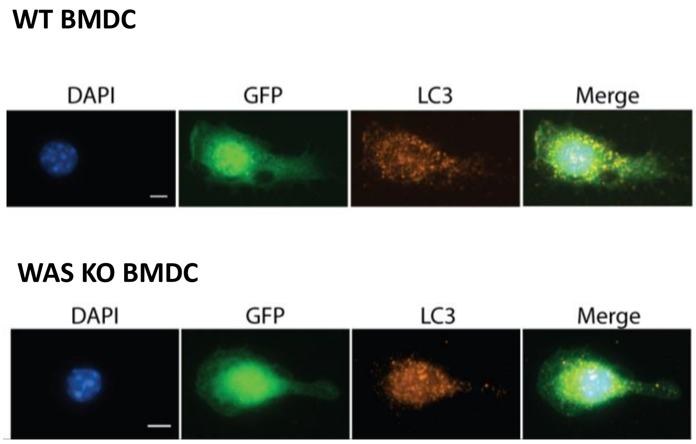

C

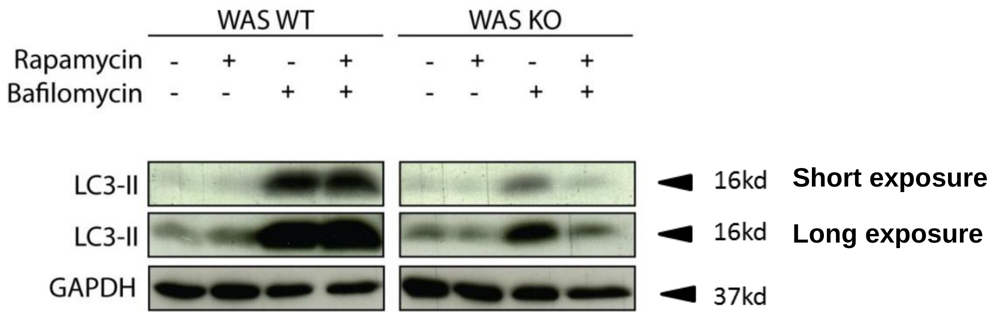

D

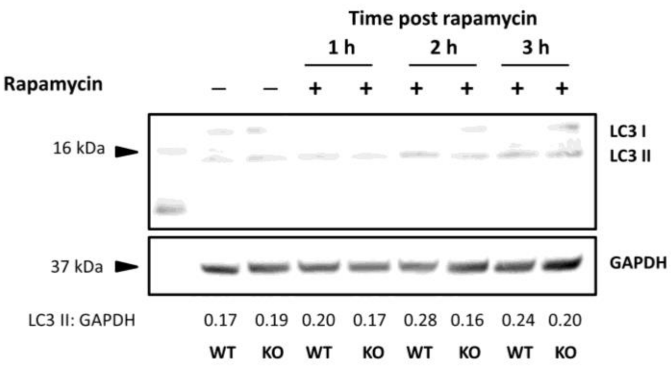

E

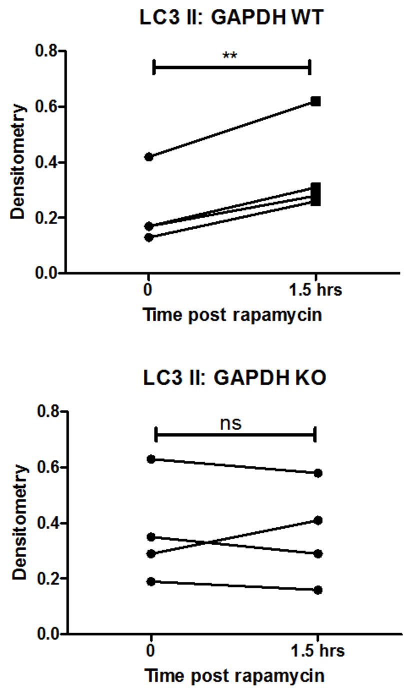

A. WT or WAS KO BMDCs were co-cultured with WT EPEC (MOI 5) at specified time-points. Post co-culture, uninfected control and infected cell lysates were immunoblotted for LC3-I and LC3-II expression. β-actin was used as loading control. A representative of 3 experiments is shown. This is the uncropped scan of the western blot shown in Fig. 5A.

B. eGFP-transduced WT and WAS KO BMDCs were fixed and stained for nuclei (DAPI), eGFP (green), and LC3 (orange). Images were taken at 63x magnification. Scale bar = 5 µm.

C. WT and WAS KO BMDCs were exposed to 50nM rapamycin and 160nM bafilomycin individually or in combination. 2 h post-stimulation, cell-lysates were subjected to 15% SDS-PAGE followed by immunoblotting for LC3 expression. GAPDH expression served as loading control. A representative blot, with short and long exposure times from 3 experiments is shown.

D. PMA-differentiated WT and WASp KO THP-1 cells (0.5 x 10^6) were exposed to 50nM rapamycin for varying times. Post-stimulation, cell-lysates were subjected to 4-12% SDS-PAGE followed by immunoblotting for LC3 expression. GAPDH expression served as loading control. A representative blot from 4 experiments is shown.

E. Densitometric analyses of 4 individual experiments showing significant LC3 conversion in WT THP-1 but not in WAS KO THP-1 cells in response to rapamycin.

Supplementary Figure 6

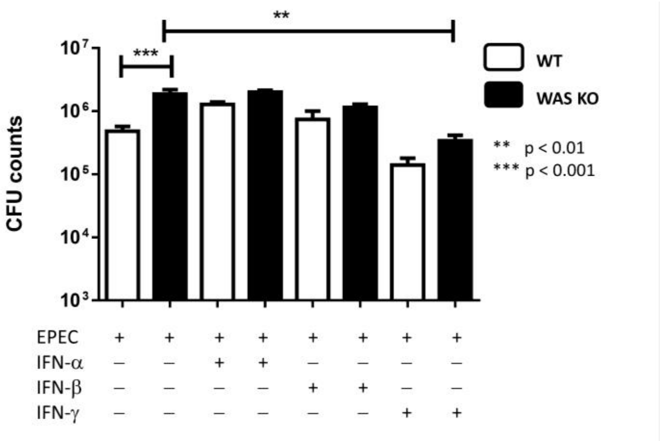

WT and WAS KO BMDCs were stimulated with IFN-α, IFN-β or IFN-γ (500U/ml) for 16 h followed by co-culture with EPEC at MOI 5 for 3 h. Intracellular bacterial survival was measured by CFU enumeration, obtained after gentamicin treatment for 2 h. Results are presented as mean CFU counts ± SEM obtained from three independent experiments done in duplicate.
