# Supplementary material for: Wiskott-Aldrich syndrome protein regulates autophagy and inflammasome activity in innate immune cells
Source: Nat Commun. 2017 Nov 17;8:1576. doi: 10.1038/s41467-017-01676-0 (PMC5691069; doi:10.1038/s41467-017-01676-0)
Supplement: Supplementary file 2 — Description of Additional Supplementary Files [file 41467_2017_1676_MOESM2_ESM.pdf]

Description of Additional Supplementary Files

File Name: Supplementary Movie 1
Description: 3-dimensional reconstruction of septin cage in wild-type murine bone marrow derived dendritic cells, showing recruitment of F-actin and septin 2 to EPEC

File Name: Supplementary Movie 2
Description: 3-dimensional reconstruction of septin cage in WAS KO murine bone marrow derived dendritic cells, showing lack of recruitment of F-actin and septin 2 to EPEC
